# COVID-19 and Work–Family Conflicts in Germany: Risks and Chances Across Gender and Parenthood

**DOI:** 10.3389/fsoc.2021.780740

**Published:** 2022-01-05

**Authors:** Mareike Reimann, Eileen Peters, Martin Diewald

**Affiliations:** Faculty of Sociology, Bielefeld University, Bielefeld, Germany

**Keywords:** work-family conflict, COVID-19, gender, parenthood, childcare, working from home, Germany

## Abstract

The COVID-19 pandemic has dramatically affected everyone’s daily life in one way or another, requiring a re-negotiation of existing strategies for work–life integration, not only for individuals but also within families and partnerships. To contribute to existing knowledge on work-life integration during COVID-19 in Germany, we look at gender and parenthood differences in the experiences of work-to-family (WFC) and family-to-work (FWC) conflicts. By accounting for employees’ previous conflict experiences, we were able to reveal the extent to which the current conditions contributed to differences in these conflicts. Moreover, we explored the relevance of demands and resources in the family and work spheres as a way to explain different levels of WFC and FWC across gender and parenthood. Our analyses are based on a sample of 660 employees from a German linked employer–employee panel study and a COVID-19 follow-up survey conducted in late 2020. Results revealed that work–family conflict experiences before the pandemic play an important role in current conflict perceptions. Whereas WFC were more likely to be accentuated during the pandemic, prior FWC experiences may have helped to mitigate conflicts under these new conditions. Work–family conflicts in general have increased during the COVID-19 pandemic, but this finding applied only to conflicts in the family-to-work direction. Although such increases were not limited to parents, they were particularly high in this group. Overall, gender differences in work–family conflicts were absent, but differences were found between mothers and fathers. The need to compensate for a lack of external childcare, as well as having to work from home, increased FWC, especially among fathers. This study suggests that FWC in particular became more important during the pandemic; however, parents were not the only ones who were disadvantaged when it came to work–life integration; childless individuals likewise struggled to balance the demands of work and private life.

## 1 Introduction

The COVID-19 pandemic has dramatically affected the daily lives of everyone in one way or another, forcing a re-negotiation of existing strategies for work–life integration. This need to revise such strategies has affected not only individuals but also families and partnerships. In Germany, since the start of the pandemic, families with children seem to have been hit exceptionally hard as a result of measures designed to protect against COVID-19 infections (Müller et al., 2020; Bujard et al., 2021). As early as March 2020, external childcare facilities were closed, and childcare support by family members (especially grandparents) or friends was limited owing to rigorous contact restrictions. Most parents then had to compensate for this lack of childcare opportunities by caring for their children at home, with mothers taking on the bulk of additional care demands (Collins et al., 2020; Kohlrausch and Zucco, 2020; Möhring et al., 2020; Müller et al., 2020; Zoch et al., 2021a). Consequently, employed parents (particularly mothers) experience difficulties in their work-life integration, leading to decreased satisfaction in both spheres (Zoch et al., 2021b; Huebener et al., 2021; Möhring et al., 2021). Although we acknowledge the disadvantaged position of parents and mothers in particular during this pandemic, we argue that existing studies may be too simplified, specifically in terms of the mechanisms in the work–life interface and the longitudinal perspective regarding previous conflict experiences.

To investigate the interplay of work and family life during the pandemic, as well as potential differences according to gender and parenthood, we refer to the well-established concept of work–family conflicts. This concept assumes that the respective demands of a person’s work life and private life compete for that individual’s time and energy and that attempts to reconcile these various life-sphere demands may lead to inter-role conflicts (Greenhaus and Beutell, 1985). Conflicts may occur when participation in the work role makes it more difficult to meet the requirements of the family role—that is, in the direction of work-to-family conflicts (WFC) — but also vice versa in the direction of family-to-work conflicts (FWC) (Gutek et al., 1991; Netemeyer et al., 1996).^1^ Most studies undertaken during the COVID-19 pandemic in Germany have focused on the individual’s satisfaction with work and family life, but information about work–family conflict during these unprecedented times is scarce. The first descriptive evidence concerning work–family conflicts suggests that there have been no gendered parenthood differences in the increase of WFC and FWC during the pandemic in Germany (Buschmeyer et al., 2021). Research from Canada suggests that childless employees and parents with older children experience lower rates of WFC compared with pre-COVID-19 levels (Schieman et al., 2021), again with no gender differences. Hence, a comprehensive analysis of WFC and FWC differences during the pandemic is still missing.

To add to the existing literature, we offer four main contributions: First, we consider WFC and FWC as different directions of possible conflicts. This is necessary given the fact that the pandemic has led to far-reaching changes in the situations at work and at home, thus increasing possible sources of conflict in both life spheres. Second, we focus on the intersection of gender and parenthood as one of the most salient topics in both scientific and public discussions about pandemic-induced strains. Differences between these groups are being comprehensively studied in terms of workloads and subjective evaluations but not in terms of work–family conflicts. Third, we investigate the association between work–family conflicts experienced during the pandemic and those experienced previously, thus providing points of departure for our current observations. Accounting for employees’ previous conflict experiences will reveal to what extent conditions during COVID-19 are contributing to differences in conflict experiences. Fourth, we explore to what degree demands and resources in the spheres of work and family are relevant for explaining different levels of WFC and FWC across gender and parenthood.

First, the most important advantage of focusing on WFC and FWC is that they directly map reconciliation conflicts in the interplay of work and family life instead of looking at work or family satisfaction separately. In line with the assumptions concerning chronic strain, work–family conflicts can be understood as the relatively enduring conflicts or threats that individuals face in their daily lives (Pearlin, 1989). Conflicts may be accepted as unavoidable concomitants of striving to meet ambitious goals in both life spheres—that is, such conflicts do not necessarily lead to decreased satisfaction or reduced overall well-being (see, e.g., Barnett and Hyde, 2001; Grönlund and Öun, 2010). The distinction between WFC and FWC is important because their antecedents and consequences differ significantly (Kossek and Ozeki, 1999; Byron, 2005). Although levels of WFC tend to be greater than those of FWC (Gutek et al., 1991; Seery et al., 2008; Nohe et al., 2015), this is not necessarily the case in a situation like the pandemic. It is reasonable to assume that a chaotic and highly demanding situation at home for which nobody could have been prepared may be more salient than working conditions.

Second, quite similar to the results of Anglo-American research, evidence from studies in Germany confirms that large parts of the German workforce experience WFC and FWC (Steinmetz et al., 2008; Gallie and Russell, 2009; Beham and Drobnič, 2010; Pausch et al., 2016; Reimann et al., 2017; Abendroth and Reimann, 2018). Still, the experience of WFC and FWC is hardly universal, meaning that whether and to what extent conflicts are experienced systematically differ between social groups, such as between parents and childless individuals and between women and men (Voydanoff, 2002). The consensus is that parents have higher levels of WFC and of FWC than do childless employees (Winslow, 2005; Innstrand et al., 2010), although there is no clear evidence for systematic gender differences despite a slight tendency for higher levels of conflict among women (Duxbury and Higgins, 1991; Frone et al., 1992b; Nomaguchi, 2009).

Third, we emphasize that work–family conflicts are not genuine experiences to the COVID-19 crisis. Rather, they build upon experiences of such conflicts before the pandemic and should consequently be analyzed in face of the pre-COVID-19 situation in mind. Nevertheless, the direction in which preexisting conflicts impinge on conflicts during the pandemic is not clear. On the one hand, life course theories about cumulative advantage and disadvantage (Crystal and Shea, 1990; O’Rand, 1996) assume that high levels of strain and conflict that were present before COVID-19 may lead to comparably higher work–family conflicts during this pandemic. Accordingly, for this group of employees, comparably high levels of conflict indicates a higher vulnerability to increased pandemic-specific demands and fewer resources available. On the other hand, a competing theoretical perspective assumes that past adverse experiences can help employees deal more successfully with the current challenges, since these individuals and couples have learned how to cope with conflict-triggering situations (Seery, 2011; Seery et al., 2013). Thus, if individuals experienced work–family conflicts before COVID-19, they may be better able to deal with the increased challenges during the pandemic, presuming that the level of conflict at the outset was not already overwhelmingly high.

Fourth, we consider that gender and parenthood differences in WFC and FWC might be explained, at least to some degree, by compositional differences in work and family demands and resources. An impressive number of studies have shown that WFC is mostly explained by work conditions, whereas FWC is predicted by family conditions (Frone et al., 1997; Kinnunen and Mauno, 1998; Byron, 2005). Moreover, it is also well-known that favorable resources and disadvantageous demands are unequally distributed across gender and parenthood status. For example, women spend more time than men do on domestic work, and mothers spend more time on childcare than fathers do, which also seems to be the case during the pandemic (Adams-Prassl et al., 2020; Kohlrausch and Zucco, 2020; Hank and Steinbach, 2021; Hipp and Bünning, 2021). In the work domain, men are more likely to have access to job autonomy and flexibility options, while women and parents receive more work–family support than do childless individuals (Den Dulk and Peper, 2007). In our study, we do not want to replicate the comprehensive evidence of several work and family resources and demands (for overviews see, e.g., Michel et al., 2011; Bakker and Demerouti, 2017; Lesener et al., 2018), but focus on two aspects of concern that are specific to the pandemic: working from home and limited childcare opportunities. Moreover, we include demands (such as increased overtime) and resources (such as job autonomy and family supportiveness on the part of the employer and co-workers) in the work domain because these are well-established predictors of work–family conflicts (Allen et al., 2013; Abendroth and Reimann, 2018) and have so far been neglected in studies during the pandemic.

## 2 Gender and Parenthood Differences in Work–Family Conflicts During COVID-19

### 2.1 The COVID-19 Pandemic in Germany

As in most other countries of the Global North, curbing the spread of COVID-19 during the first wave of the pandemic became a political and societal priority starting in mid-March 2020. As part of the government’s “hard lockdown” strategy, schools and childcare facilities were closed immediately, being available only for parents who had specific, so-called “system-relevant” occupations (e.g., health sector workers and employees responsible for ensuring basic supply chains). Simultaneously, working from home became a reality for many employees as another measure to reduce the risk of infection by limiting contact with others. After decreasing infection numbers, these restrictive measures were partly loosened beginning in May and throughout the summer. Nevertheless, the childcare situation in particular remained tense. In response to the rapidly rising infection numbers from October 2020 on, the German government re-enforced pandemic measures. Since the data used for our study were collected from October to December 2020, the pandemic situation and the infection protection measures during that period need to be especially considered. After gradually restricting social activities in October and November, the government imposed another lockdown in November 2020. Consequently, retail stores were closed again, private contacts were limited to an absolute minimum of two households, and employers were obligated to make working from home available whenever possible.

For many individuals, these measures increased the potential for problems associated with social isolation and psychological disorders (Lengen et al., 2020; Rudolph et al., 2021) but especially brought employed parents and their children in a situation of overburdening since they had to work at home and simultaneously supervise their children. Although schools and daycare facilities generally remained open, most schools adopted hybrid models, which meant a combination of homeschooling and teaching at school. The schedules varied (e.g., alternating weeks or days), with changes often announced only 1 day in advance or even on the morning of the change. Schools and childcare facilities needed to be closed again if any infections were detected among children or teachers. Moreover, because personal contacts were restricted, private and informal childcare was much less available than it was before the pandemic. Thus, most parents had to face an unstable care situation, having to switch between open care facilities and having to care for their children at home (for a comprehensive and detailed review of pandemic measures in Germany, see Bujard et al., 2021). These conditions challenged parents’ practiced routines and arrangements of work–family reconciliation.

### 2.2 Accounting for Prior Work–Family Conflict Experiences: Accentuation or Learning?

The COVID-19 pandemic can generally be viewed as a life event stressor (Pearlin et al., 1981; Pearlin, 1989), as it is an unexpected and uncontrolled event in the life course of all people. The sudden emergence of the pandemic situation with all its restrictions and hardships to deal with, forced processes of role-restructuring and the need to adapt daily life practices. As a result, individuals were challenged to revise daily routines and re-negotiate arrangements in certain areas of their lives (Buschmeyer et al., 2021), leading inevitably to new inter-role conflicts. Thus, during the pandemic, work–family conflicts—both FWC and WFC—were bound to arise or increase.

First descriptive evidence concerning work–family conflicts in Germany suggests that WFC and FWC did indeed increase during the pandemic (Buschmeyer et al., 2021), but it is hardly researched how these conflict experiences differ between social groups. Based on previous research related to the household division of labor and employees’ satisfaction with work and family, it can be expected that the main lines of differentiation for work–family conflicts will likewise be parenthood and gender. However, descriptive analyses found no gendered parenthood differences in Germany (Buschmeyer et al., 2021). Furthermore, research from Canada suggests that childless employees and parents with older children have experienced even lower WFC during the pandemic than they did pre-COVID-19 (Schieman et al., 2021), again with no gender differences.

We argue that work–family conflicts during the pandemic cannot be adequately understood without taking the levels of pre-COVID-19 conflicts into account. Only then, the specific contribution of the pandemic to the conflict level experienced during the pandemic can be assessed. Nevertheless, how the levels of conflicts that were present prior to the pandemic are associated with the degree to which individuals experience WFC and FWC in light of the protective measures instituted to fight infections remains an open question. If employees who had difficulty integrating high work and family demands and/or lacked the resources to handle these demands experienced massive conflicts before the pandemic, the superimposition of COVID-19-related demands may have simply exacerbated these pre-existing strains. According to the assumptions regarding cumulative disadvantages (Crystal and Shea, 1990; O’Rand, 1996), individuals who are already disadvantaged will be at higher risk of disadvantage in the future—that is, disadvantages will be accentuated over time. Those who already had higher pre-pandemic levels of conflict would be expected to experience even more conflicts during the pandemic. Hence, we derive our first hypothesis.

Hypothesis 1 The higher the level of conflicts prior to the COVID-19 pandemic, the greater the conflicts during the pandemic (accentuation hypothesis).

An alternative theoretical perspective assumes that facing and managing difficulties in life can have benefits for handling future difficulties (Seery, 2011). Individuals who are exposed to limited adverse experiences may foster subsequent resilience toward future adversity, since they acquire coping strategies and learn to handle those situations more effectively (Seery et al., 2010). They will perceive stressful situations in general as being more manageable (rather than overwhelming) and are thus better able to deal with them in the future. However, it has been suggested that those learning processes will not be successful if the experienced strain is either too low or too high, suggesting a U-shaped relationship between previous adverse experiences and current experiences (Seery, 2011; Seery et al., 2013). In the case of work–family conflicts, this view indicates that if individuals experienced low or very high pre-COVID-19 levels of WFC or FWC, they should experience higher levels of conflicts during the COVID-19 pandemic. In the case of high levels of conflicts, the argument is similar to the accentuation hypothesis: if the extent of an experienced stressor is too great, individuals will be overwhelmed and thus they are not able to learn from their experiences. In the case of low levels of conflicts, the stress induced by the conflict is not enough to trigger the need to learn how to deal with it effectively, so that coping strategies will not be available in future situations. Therefore, our second hypothesis, the learning hypothesis, suggests that individuals with no or very high pre-COVID-19 levels of work–family conflicts will experience more conflicts during the COVID-19 pandemic. In contrast, those with moderate pre-COVID-19 levels of conflicts will experience similar or even lower levels during the pandemic. Thus, employees who experienced moderate levels of conflicts before the pandemic should benefit from their prior experiences handling conflicts and should therefore experience lower levels of conflicts during the COVID-19 pandemic.

Hypothesis 2 Individuals who have very high levels of conflict (resulting from overload) or, in contrast, have no work–family conflicts (with no need to accommodate conflicts) before the COVID-19 pandemic will experience more conflicts during the pandemic, whereas those with moderate pre-COVID-19 levels will experience similar or even lower levels of conflicts when compared with pre-COVID-19 levels (learning hypothesis).

### 2.3 Gender and Parenthood Differences in Work–Family Conflicts: The Role of Family and Work Demands and Resources

The various social groups within the workforce face different demands and dispose of diverse resources, thus experiencing work–family conflicts in different ways. A large body of literature has focused on these work–family conflicts differences, providing evidence for the relevance of almost every work or family demand or resource under study (for reviews, see Michel et al., 2011; Bakker and Demerouti, 2017; Lesener et al., 2018). Working conditions either can be perceived as demanding and can increase conflicts, or they can be used as resources to mitigate demands or to directly reduce strain (Bakker et al., 2005). Similarly, family characteristics either can be highly demanding in terms of time, emotional impact, and/or cognitive stress (e.g., greater responsibilities regarding childcare or family involvement) or can provide material resources or emotional support (Frone et al., 1992a). One important finding in earlier research is that, overall, work demands and resources tend to be more predictive of WFC, whereas family demands and resources tend to be more predictive of FWC (Frone et al., 1997; Kinnunen and Mauno, 1998; Byron, 2005).

Gender, parenthood, and the intersection of these two characteristics are among the most investigated topics in studies of the work–life interface (Parasuraman and Greenhaus, 2002; Bianchi and Milkie, 2010). Results of such research have shown that these groups represent important lines of demarcation in the overall distribution of conflicts, which are defined by the typical differences in family and work demands and resources (Voydanoff, 2002). In the following section, we will briefly refer to the work and family demands and resources that generally differ across gender and parenthood and thus result in differences in these groups’ experiences of work–family conflicts. However, our main focus is on gender and parenthood differences specifically in relation to pandemic-induced demands and resources, as well as on implications of those demands and resources for the experience of work–family conflicts. As we will explain, we see these specific demands in childcare in the family domain, and increased overtime in the work domain. Resources are working from home, job autonomy, and family-supportiveness of the workplace in the work domain.

#### 2.3.1 Family Demands and Resources

Overall, parents are expected to encounter more demands than childless individuals do because of their greater responsibilities as a part of family life; thus, they experience greater conflicts (Nomaguchi, 2009). Indeed, existing research is quite consensual in finding that working parents have higher levels of WFC and FWC when compared with workers who have no children (Winslow, 2005; Innstrand et al., 2010). In direct comparison, despite the fact that fathers are spending more time with their children than before the pandemic (Bianchi et al., 2000; Sandberg and Hofferth, 2001; Gauthier et al., 2004), mothers still shoulder most of the care demands. Consequently, studies have found that mothers have higher levels of WFC and FWC when compared both with fathers and with childless women and men (Duxbury and Higgins, 1991; Gutek et al., 1991; Nomaguchi, 2009). In particular, having younger children is associated with higher WFC and FWC levels because of the time-consuming care investments (Gallie and Russell, 2009; Martinengo et al., 2010; Allen and Finkelstein, 2014). Although having institutionalized childcare available does help to prevent WFC and FWC for all parents, this resource has proved to be especially important for mothers (Reimann et al., 2019).

As previously addressed, a major issue during the COVID-19 pandemic concerns the instability, or complete lack, of formal and informal childcare. During lockdowns, external childcare options are limited by the closing of schools, kindergartens, and other childcare facilities; even when childcare facilities are generally open, day-to-day childcare is unpredictable because of the changing quarantine regulations. In addition, informal childcare, especially as provided by grandparents or friends, may be limited owing to rigorous contact restrictions and the urgent request to protect the elderly, who are at greater risk for more fatal COVID-19 infections. As a result, parents face greater childcare demands, may it be looking after their small children at home or supervising older children’s schoolwork. Compared with childless individuals, parents, especially those with young children, are more likely to be burdened with increased demands as part of family life during the pandemic. More precisely, employed parents have time-sensitive demands in the family domain because childcare is necessary throughout the day. Therefore, it is likely that FWC levels are higher for all parents than for non-parents, as being bound to childcare obligations directly affect and restrict the time and energy parents need to meet their work obligations. Conversely, parents most likely experience higher levels of WFC as well, because they have only limited leeway when it comes to handling work issues as their children spend more time at home and need supervision that cannot be postponed or outsourced because external childcare options are limited or sometimes not available at all:

Hypothesis 3 Parents experience higher levels of FWC and WFC than do non-parents, even when controlling for pre-COVID-19 conflicts.

Nevertheless, one positive outcome of this situation should not be neglected: being bound to the home because of contact restrictions, less opportunities for activities outside the home, working from home or even quarantine, enables families to spend more time together. Even though increased family time under these pandemic conditions can be a source of conflicts, it can also foster family interactions, improve the quality of parent–child relationships, and allow for emotional closeness, especially for mothers and fathers who work full-time (Russell et al., 2020; Spinelli et al., 2020; Feinberg et al., 2021; Mantovani et al., 2021).

Studies have shown that mothers tend to take on the lion’s share of the additional childcare demands by increasing the time they spend on childcare, homeschooling, and domestic work (Adams-Prassl et al., 2020; Kohlrausch and Zucco, 2020; Hank and Steinbach, 2021; Hipp and Bünning, 2021). Compared with the pre-COVID-19 period, fathers are also contributing more by providing unpaid labor at home; however, mothers continue to be the primary caregivers and fathers remain in a supporting role (Zoch et al., 2021b; Kreyenfeld and Zinn, 2021). When viewed from this angle, it would seem that mothers now experience even more work–family conflicts than fathers do, even when we control for the pre-pandemic situation, since the added burden would increase the already high demands placed on mothers. However, we argue that fathers are particularly affected by the new situation at home and by the need to re-negotiate and adapt existing strategies for the division of labor. Previous arrangements often spared fathers, allowing them to focus on gainful work (Trappe et al., 2015). Hence, the fundamental changes in existing processes at home may affect fathers more than mothers, because they are less used to being involved in everyday childcare struggles and keeping things running. Family life during the pandemic may therefore impose more strain on fathers, leading to higher levels of FWC for them than for mothers when we account for pre-COVID-19 conflicts:

Hypothesis 4 Fathers experience higher levels of FWC than mothers do.

#### 2.3.2 Work Demands and Resources

Different working time arrangements are among the most widely studied work demands and resources in relation to work–family conflicts as they directly affect the time available for family matters. Long working hours (Gallie and Russell, 2009; Adkins and Premeaux, 2012), non-standard schedules (Taiji and Mills, 2020), and irregular shiftwork (Zhao et al., 2021) are demanding working time characteristics that are associated with higher WFC and FWC. Increased overtime during the COVID-19 pandemic should be associated with higher WFC and FWC for all individuals. However, as having to work longer hours intensifies the restriction of time available to compensate for the lack of childcare and increased family demands, parents should be burdened by increased overtime more strongly than non-parents.

Hypothesis 5 The positive relationship between increased overtime during the pandemic and levels of WFC and FWC is stronger for parents than for non-parents.

A recurring argument is made suggesting that different types of flexible work may be a key to solving the problem of work–life integration (Hill et al., 2010). The basic idea is that flexible work is a resource that allows employees to adapt their work and family demands to individual circumstances and thus avoid or at least reduce the risks of conflicts (Shockley and Allen, 2007; Schieman and Glavin, 2008). Flexible work can refer to flexibility in the timing of work (flex-time), flexibility in the location of work (flex-place), and/or flexibility in deciding what work to do and how to do it, also known as (job) autonomy (Baillien et al., 2011). Men are more likely than women to work in positions with higher status (Stojmenovska and England, 2021) and are therefore more likely to have greater job autonomy as well as greater negotiating power to choose from these flexibility options (Wheatley, 2017). As a result, gender differences in work–family conflicts might be explained by the fact that women have comparably fewer opportunities to engage in flexible work. However, the evidence suggests that flexible work can also be considered a demand, especially if the flexibility options serve the interests of the employer more than those of the employee (Abendroth and Diewald, 2019; Marx et al., 2021). Consequently, studies have found positive as well as negative effects on both WFC and FWC (Allen et al., 2013), especially when it comes to working from home (Allen et al., 2015).

Research conducted during the COVID-19 pandemic indicates that working from home is more frequently used by women, especially mothers, as compared to men and fathers (Kohlrausch and Zucco, 2020; Hipp and Bünning, 2021; Lanfranconi et al., 2021; Reichelt et al., 2021). Since family boundaries seem to be more permeable than work boundaries (Frone et al., 1992b), working from home during the extreme circumstances of the pandemic is likely to engender more inter-role conflicts: work and family issues are hardly separable when everyone is at home. Therefore, one would expect that WFC in particular would be affected by working from home, not only for specific groups but for all individuals.

Nevertheless, as argued above, the situation of parents with children being at home may be somewhat different from childless persons. On the one hand, working from home may be considered a benefit and might represent a resource because parents who do not have to leave home for work are better able to coordinate their additional childcare obligations and their work demands. For example, they could schedule their working hours at times of the day when the children are sleeping, or they could monitor their children playing in the next room while simultaneously carrying out their work tasks. On the other hand, negative effects of this scenario are just as likely. The constant juggling between work and childcare, the need to concentrate while children are playing loudly, and the permanent involvement in family conflicts, be it with one’s partner or one’s children, makes separating work and family almost impossible. In comparison, childless women and men do not struggle to balance childcare and work when working from home and may benefit from saving time for commuting. Though the lack of boundaries between work and family, the risk of isolation from co-workers, and a lack of cohesiveness when working permanently at home are well-known risks of working from home, these may be less salient during the pandemic when many or even all employees in similar positions work from home. Moreover, communication issues or the lack of contact with co-workers or supervisors is a problem for most people and should not disadvantage women more than men. In sum, even though the results of past studies were inconclusive regarding the relationship between working from home and work–family conflicts, we assume that parents in particular should benefit when they can transfer their work responsibilities to the home setting (Hypothesis 6a).

Similar to working from home, an ambivalent role of job autonomy is addressed in the stress of higher status hypothesis, assuming that a high level of job autonomy might be linked to greater conflicts (see, e.g., Badawy and Schieman, 2020). Nevertheless, job autonomy might be especially helpful during the COVID-19 pandemic because it allows employees to arrange work tasks more flexibly according to individual needs. For instance, complex work tasks that require more concentration could be scheduled to quieter times of the day, and work goals for the week could be adjusted according to school schedules if homeschooling is reinstituted. These resources should be beneficial for all individuals, but parents may benefit even more, especially since they may require more resources to compensate for their greater family demands (Hypothesis 6b).

In addition to flexibility with regard to the individual job, research has addressed the family-supportiveness of the workplace in the form of family-supportive workplace cultures as being related to lower levels of WFC and FWC (Abendroth and Reimann, 2018; Reimann et al., 2019). Family supportiveness provided by employers and co-workers may prevent an increase in conflicts for parents during the COVID-19 pandemic. If the employer understands parents’ family obligations and co-workers help if personal demands arise, parents might feel less conflicted when faced with the need to handle a stressful childcare situation and will thus experience lower levels of WFC and FWC (Hypothesis 6c).

Hypothesis 6 The negative relationship between job resources and levels of WFC and FWC is stronger for parents than for non-parents because parents benefit more from such resources during the COVID-19 pandemic. These resources include

a) Working from home.
b) Job autonomy as an individual resource.
c) Family-supportiveness by the employer and co-workers as organizational resources.

## 3 Methods and Data

### 3.1 Data

We tested our hypotheses using the LEEP-B3 dataset, which is based on administrative records from the German Federal Employment Agency, provided by the Institute for Employment Research (IAB), and on survey data collected by Bielefeld University (Diewald et al., 2014). The LEEP-B3 dataset currently consists of three regular waves: Wave 1 (2012/13), Wave 2 (2014/15), and Wave 3 (2018/19) and a COVID-19 follow-up survey conducted from October to December 2020 to explore how the COVID-19 pandemic affected employees’ working conditions and work–life integration. Large establishments (more than 500 workers subject to social security payments) and employees within these establishments were randomly selected to participate in the LEEP-B3 survey.^2^ In all three waves, administrative records were linked to the survey data if the respondents consented to such a linkage. A comparison of the sample with the distribution of employees and employers in the German labor market showed that the data were representative of large workplaces across industries, workplace size, region, and their workforces.^3^

Because the COVID-19 follow-up survey was conducted late in 2020, the observation period overlapped with the second surge of the COVID-19 pandemic in Germany. The gross sample for this survey incorporated all the employees who participated in the third wave of the main survey and who gave permission to be contacted again. Of all those contacted, 810 employees (15.9%) participated in the survey. After excluding employees for whom information was missing on the dependent variables (5.8%) and those with missing values on the co-variates (10.2%), the final sample for analysis consisted of 660 respondents (78 mothers, 149 fathers, 194 childless women, and 239 childless men). We link the information of the data collected during COVID-19 to information from the previous waves of the LEEP-B3 panel to investigate gender and parenthood differences in WFC and FWC during COVID-19, to account for the experience of conflicts before the COVID-19 pandemic, and to consider work and family experiences during the pandemic, especially regarding childcare and working from home.

### 3.2 Measurements

#### 3.2.1 Dependent Variables

This study explores WFC and FWC resulting in two dependent variables. To capture information about WFC, participants were asked to respond to the following two statements: “When I come home after work, I often lack the energy for private activities and commitments” and “I miss important leisure activities with my partner, family, and friends due to my time constraints.” Responses were measured on a 5-point Likert scale ranging from 0 (“Does not apply at all”) to 4 (“Applies completely”). These two items were combined in a single measure to capture overall WFC during the pandemic.^4^ The values range from 0.5 to 5, where high values indicate high levels of WFC. Using the same Likert scale, FWC is measured based on the following two statements: “Because I am often stressed from family responsibilities, I have a hard time concentrating on my work” and “Due to private appointments, I often have problems getting my work done.” These two items were likewise combined to produce a single measure for capturing overall FWC during the pandemic. The internal consistency of all dimensions was acceptable, with Cronbach’s alpha coefficient of reliability exceeding the conventional level of acceptability (WFC: *α* = 0.72; FWC: *α* = 0.68). These questions were also included in Wave 3 (2018/19), which allowed us to consider pre-pandemic levels of work–family conflicts.

#### 3.2.2 Independent Variables

The main independent variables are employees’ gender and parenthood status. Binary variables are used to measure gender (1 = women, 0 = men) and parenthood status (1 = parents, 0 = non-parents). Because care demands decrease once children grow older, parents with children older than 18 are categorized as non-parents. Overall, the sample includes 272 women, of which 194 are childless and 78 mothers, and 388 men, 239 childless and 149 fathers.^5^

Demands in private life during COVID-19 are captured by childcare responsibilities, either for preschool children (0 = no) or for the supervision of schoolwork (0 = no).

Work resources and demands are captured by working from home during the COVID-19 pandemic (0 = no), as well as the degree of job autonomy and family-supportiveness of co-workers and supervisors. Job autonomy is captured by an index of the following three items: 1) “Within my working hours, I have control over the sequence of my work activities”; 2) “I can decide how to execute my work tasks”; and 3) “I can define my job objectives.” Employees could indicate on a 5-point Likert scale if this “Does not apply at all” (=0) or “Applies completely” (=4). The job autonomy index ranges from 0 to 12, with higher values signaling greater job autonomy (Cronbach’s *α* = 0.79). Family supportiveness is measured by supervisors’ support in reconciling the employee’s work and private life and by co-workers’ help with work tasks if the employee’s private life interfered with work. Respondents were asked to rate the degree of supervisor and co-worker support on a 5-point Likert scale ranging from 0 (“Does not apply at all”) to 4 (“Applies completely”). We created two binary variables that depict whether supervisors and co-workers are less supportive (0) or highly supportive (1).^6^ As a common work demand, we included whether employees face increased overtime during the pandemic (0 = no).

More than one fourth of all fathers and mothers in our sample have preschool children at home during the pandemic and more than half of all the parents supervise their children’s schoolwork (Table 1).^7^ Fathers show the highest degree of job autonomy, whereas childless women have the lowest. With regard to family-supportiveness, there is not much variation across the observed groups, underlining the fact that it is not just parents who are supported in efforts to combine their work and private life, but childless employees receive such support as well. Finally, fathers are most likely to work from home during the pandemic, followed in decreasing order by childless men, mothers, and childless women. This differs from results of previous studies showing that women more frequently transitioned to work from home (e.g., Hipp and Bünning, 2021).

**TABLE 1:** Descriptive statistics family and work resources and demands.

	Overall (*N* = 660)		Fathers (*N* = 149)		Childless men (*N* = 239)		Childless women (*N* = 194)		Mothers (*N* = 78)		
	Mean	SD	Mean	SD	Mean	SD	Mean	SD	Mean	SD	Min/Max
Family demands											
Preschool children	0.12	0.32	0.32	0.47	0.03	0.17	0.01	0.071	0.27	0.44	0/1
Supervision schoolwork	0.20	0.40	0.55	0.50	0.02	0.154	0.01	0.101	0.55	0.50	0/1
Work resources and demands											
Worked from home during pandemic	0.68	0.47	0.81	0.39	0.70	0.46	0.55	0.499	0.66	0.48	0/1
Job autonomy index	8.35	2.57	8.75	2.21	8.38	2.41	8.01	2.997	8.39	2.53	0/12
Family-supportiveness supervisor	0.51	0.50	0.51	0.50	0.49	0.501	0.53	0.50	0.58	0.50	0/1
Family-supportiveness co-workers	0.52	0.50	0.52	0.50	0.50	0.50	0.54	0.50	0.51	0.50	0/1
Increased overtime during pandemic	0.12	0.32	0.12	0.33	0.10	0.30	0.14	0.35	0.10	0.30	0/1

##### Controls

All multivariate models include controls for employees’ socio-demographic, human capital, and employment characteristics. These incorporate having a partner (0 = no) and the employees’ age. A categorical variable is included for age of the youngest child (0 = ages 0 to 3, 1 = ages 4 to 10, or 2 = ages 11 to 18). Controlling for income (monthly gross income, logarithmic) is crucial because it indirectly captures living conditions such as quiet neighborhoods, available living space (e.g., garden access or separate working space), and internet access (e.g., Hoffmann et al., 2003). Moreover, a higher income directly influences possibilities for additional childcare opportunities, leisure activities, and health care to compensate for work and family demands (Jäckel and Wollscheid, 2007; Kreyenfeld and Krapf, 2016). Thus, we consider a higher income to be a helpful resource in mitigating work–family conflicts in both directions as we do not have information on the actual availability of additional childcare opportunities during the COVID-19 pandemic. Furthermore, models include information about whether employees hold a university degree (0 = no), whether they have supervisory responsibilities (0 = no), whether they have experience in working from home (0 = no), and their contracted working hours.

Overall, 8% of the participants have children aged 0 to 3, 11% have children aged 4 to 10, and 15% have children aged 11 to 18 (Table 2). Fathers are most likely to be supervisors (45%), whereas only 12% of mothers have supervisory responsibilities. Childless women are least experienced in working from home, and fathers are most experienced. 12% of the participants faced increased overtime during the pandemic, almost no employees reduced their working hours, and there is only marginal variation across the observed groups.

**TABLE 2:** Descriptive statistics control variables.

	Overall (*N* = 660)		Fathers (*N* = 149)		Childless men (*N* = 239)		Childless women (*N* = 194)		Mothers (*N* = 78)		
	Mean	SD	Mean	SD	Mean	SD	Mean	SD	Mean	SD	Min/Max
Partner	0.86	0.35	0.97	0.18	0.82	0.39	0.83	0.38	0.88	0.33	0/1
Age	47.63	8.97	46.04	7.29	49.00	9.79	48.74	9.27	43.86	6.83	22/58
Children 0–3 years	0.08	0.27	0.24	0.43	—	—	—	—	0.18	0.39	0/1
Children 4–10 years	0.11	0.32	0.33	0.47	—	—	—	—	0.34	0.48	0/1
Children 11–18 years	0.16	0.36	0.44	0.50	—	—	—	—	0.48	0.50	0/1
Monthly gross income (log.)	8.40	0.49	8.66	0.42	8.57	0.41	8.16	0.44	8.00	0.42	6.75/10.28
University degree	0.58	0.49	0.67	0.47	0.59	0.49	0.45	0.50	0.7	0.46	0/1
Supervisory position	0.30	0.46	0.45	0.50	0.34	0.48	0.21	0.41	0.12	0.33	0/1
Experience working from home	0.40	0.49	0.56	0.50	0.40	0.49	0.27	0.45	0.41	0.50	0/1
Contracted working hours	36.15	6.00	38.04	3.56	38.57	2.65	34.25	6.62	29.88	8.81	10/48

### 3.3 Analytical Strategy

To test our hypotheses, we estimate linear regression models with clustered robust standard errors at the workplace level to at least partially account for employees clustering in workplaces.^8^ The first analysis step is to explore differences in WFC and FWC in relation to gender, parenthood, and the intersection of the two. Second, in order to examine whether pre-pandemic experiences in work–family conflicts predict greater or lesser conflicts during the pandemic, we include WFC and FWC measured in the pre-COVID-19 wave. To capture nonlinearity in the relationship between previous conflict experiences and work–family conflicts during the pandemic, we include interacted pre-COVID-19 WFC and FWC, respectively. Third, in order to analyze whether varying work and family conditions explain differences in WFC and FWC, we add work and family demands and resources to the predictions. Fourth, in order to test whether mothers and fathers differ in the relationship between family demands and work–family conflicts, we estimate separate models for parents only. Here, we incorporate a categorical variable, which combines information on gender and childcare responsibilities. Finally, in order to analyze whether work resources and demands affect parents and non-parents differently, we add two-way interaction terms between parenthood status and increased overtime during the pandemic, working from home during the pandemic, job autonomy, and family supportiveness of supervisors and co-workers.

## 4 Results

### 4.1 Descriptive Results

Table 3 displays the comparisons of WFC and FWC before and during the pandemic, each for the whole sample, men, women, non-parents, and parents. Table 4 shows the WFC and FWC comparisons for fathers, childless men, childless women, and mothers. Overall, there was a significant increase in FWC during the COVID-19 pandemic (*diff* = 0.09; *p* < 0.05). Looking at specific groups reveals that men’s FWC significantly increased during the pandemic (*diff* = 0.12; *p* < 0.05), whereas for women, there is no significant difference in FWC. Parents and non-parents both experience increased FWC, although the difference compared with pre-pandemic FWC is only marginally significant (*diff*_*parents*_ = 0.12; *p* < 0.10, *diff*_*non-parents*_ = 0.07; *p* < 0.10). In contrast, we do not detect an overall difference in WFC compared with the pre-COVID-19 period. However, men’s WFC decreased (*diff* = -0.07; *p* < 0.10), although this difference is statistically significant only at the 90% significance level.

**TABLE 3:** Descriptive statistics overall, gender, and parenthood.

	Overall (*N* = 660)		Men (*N* = 388)		Women (*N* = 272)		Non-parents (*N* = 433)		Parents (*N* = 227)	
	Mean	SD	Mean	SD	Mean	SD	Mean	SD	Mean	SD
FWC 2018/19	1.57	0.66	1.56	0.64	1.60	0.69	1.54	0.67	1.64	0.64
FWC 2020	1.66	0.73	1.68	0.74	1.63	0.71	1.60	0.69	1.77	0.79
Change	0.09*	0.03	0.12*	0.04	0.03	0.05	0.07^+^	0.04	0.12^+^	0.05
WFC 2018/19	2.60	0.97	2.59	0.95	2.61	0.99	2.57	0.97	2.66	0.95
WFC 2020	2.54	1.00	2.51	0.97	−2.59	1.02	−2.50	0.99	−2.63	0.98
Change	−0.06	0.04	−0.07^+^	0.05	0.02	0.06	0.07	0.04	0.03	0.06

Note: ^+^ *p* < 0.10, *p < 0.05, **p < 0.01, ***p < 0.001.

**TABLE 4:** Descriptive statistics intersection gender and parenthood.

	Fathers (*N* = 149)		Childless men (*N* = 239)		Childless women (*N* = 194)		Mothers (*N* = 78)	
	Mean	SD	Mean	SD	Mean	SD	Mean	SD
FWC 2018/19	1.61	0.61	1.53	0.66	1.55	0.69	1.70	0.70
FWC 2020	1.79	0.84	1.62	0.67	1.59	0.72	1.72	0.70
Change	0.18**	0.07	0.09^+^	0.05	0.03	0.06	0.02	0.08
WFC 2018/19	2.72	0.94	2.51	0.96	2.65	1.00	2.53	0.98
WFC 2020	2.67	0.96	2.42	0.97	2.60	1.02	2.55	1.02
Change	−0.05	0.07	−0.09	0.06	−0.05	0.07	0.02	0.12

Note: ^+^ *p* < 0.10, *p < 0.05, **p < 0.01, ***p < 0.001.

Fathers experience particularly increased FWC during the pandemic (*diff* = 0.18; *p* < 0.01). Childless men’s FWC likewise increased, though to a lesser extent, and the difference is only marginally significant (*diff* = 0.09; *p* < 0.10). In contrast, neither childless women’s FWC nor mothers’ FWC are significantly different from their pre-pandemic levels of FWC. Moreover, there are no statistically significant differences in WFC for any of the observed groups.

### 4.2 Multivariate Results

#### 4.2.1 Accentuating or Learning?

**FIGURE 1:**
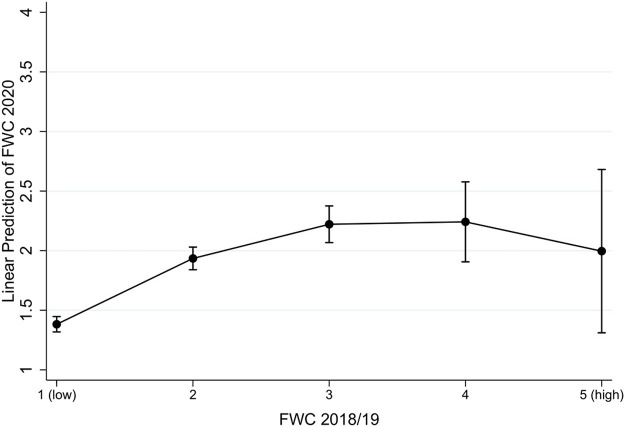
Relationship Pre-COVID-19 Family-Wok Conflicts with Family-Work Conflicts during the Pandemic. Note: Results based on Model M1, in Supplementary Appendix Table SA2. 95% confidence bands for one-tailed tests.

Hypotheses 1, and 2 assert that work–family conflict experiences before the COVID-19 pandemic predict differences in WFC and FWC during the pandemic. Figure 1 displays the relationship between previous FWC and pandemic FWC (comprehensive models can be found in Supplementary Appendix Table SA2). The non-linear relationship is a reversed U-shape, suggesting that employees who had low levels of FWC before the pandemic also have fewer conflicts during COVID-19. However, if pre-pandemic levels of conflicts exceeded a certain threshold (medium-level), higher levels of conflicts predict lower levels of FWC during the pandemic. Thus, the non-linearity assumption of Hypothesis 2 (learning hypothesis) is supported, but in a reversed way.

**FIGURE 2:**
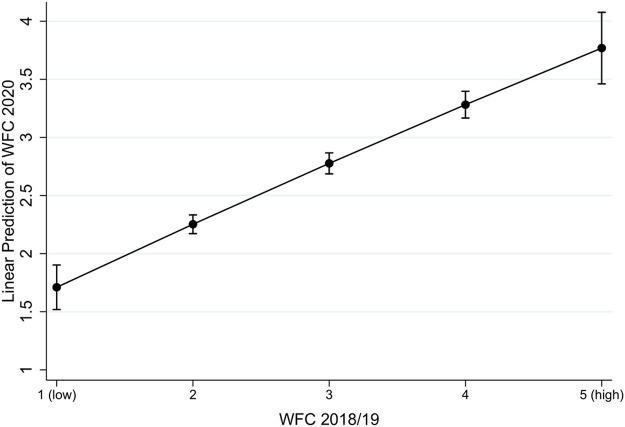
Relationship Pre-COVID-19 Work-Family Conflicts with Work-Family Conflicts during the Pandemic. Note: Results based on Model M2, in Supplementary Appendix Table SA2. 95% confidence bands for one-tailed tests.

Figure 2 displays the relationship between WFC before and during the COVID-19 pandemic. In contrast to FWC, the results show a linear relationship between pre-pandemic conflicts and WFC during the pandemic. Hence, the higher the levels of WFC before the pandemic, the higher the experienced WFC during the pandemic, thus supporting Hypothesis 1 (accentuating hypothesis).

#### 4.2.2 Gender and Parenthood Differences

The following analyses focus on gender, parenthood, and intersectional gender/parenthood differences in WFC and FWC to test Hypotheses 3, and 4. As shown in Table 5, women experience lower levels of FWC during the pandemic than men did, though this difference is statistically significant only at the 90% significance level (*b* = -0.095; *p* < 0.10). Contradicting Hypothesis 3 stating that parents should experience greater conflicts than non-parents, we do not detect a significant difference between those groups. Hence, parents and non-parents perceive similar conflicts during the pandemic when pre-COVID-19 conflict levels are accounted for. However, the following analyses will further clarify that it is not parenthood per se but the age of the children that predicts differences in work–family conflicts.

**TABLE 5:** Linear regressions on employees’ FWC and WFC by gender, parenthood, and the intersection of gender and parenthood (*N* = 660).

	Gender and parenthood		Intersection gender/parenthood	
	FWC (M1)	WFC (M2)	FWC (M3)	WFC (M4)
Women	−0.095^+^	0.069		
	(0.056)	(0.089)		
Parents	−0.047	0.110		
	(0.074)	(0.088)		
Gender and parenthood (Ref. Fathers)				
Childless men			−0.017	−0.146
			(0.093)	(0.101)
Childless women			−0.067	−0.052
			(0.100)	(0.114)
Mothers			−0.221*	0.000
			(0.097)	(0.157)
Partner	−0.009	−0.118	−0.016	−0.122
	(0.068)	(0.111)	(0.068)	(0.113)
Age	−0.011**	−0.006	−0.011**	−0.006
	(0.004)	(0.004)	(0.004)	(0.004)
Age children (Ref. Children 13–18)				
Children 0–3	0.253^+^	0.147	0.240^+^	0.140
	(0.135)	(0.148)	(0.137)	(0.151)
Children 4–12	0.110	−0.067	0.109	−0.067
	(0.117)	(0.119)	(0.116)	(0.119)
Monthly gross income (log.)	−0.130^+^	−0.025	−0.139*	−0.030
	(0.071)	(0.106)	(0.070)	(0.104)
University degree	0.093	−0.003	0.103^+^	0.003
	(0.057)	(0.077)	(0.055)	(0.077)
Supervisory position	0.037	0.030	0.032	0.027
	(0.059)	(0.079)	(0.058)	(0.080)
Experience working from home	0.081	−0.130^+^	0.084	−0.129^+^
	(0.054)	(0.069)	(0.053)	(0.069)
Contracted working hours	0.001	0.007	0.000	0.006
	(0.005)	(0.008)	(0.005)	(0.008)
FWC 2018/19	0.370***		0.372***	
	(0.055)		(0.055)	
WFC 2018/19		0.516***		0.515***
		(0.032)		(0.033)
Constant	2.575***	1.532^+^	2.695***	1.737*
	(0.549)	(0.787)	(0.567)	(0.797)

Note: Standard errors in parentheses; ^+^ *p* < 0.10, *p < 0.05, **p < 0.01, ***p < 0.001.

Looking at the intersection of gender and parenthood reveals that mothers have lower levels of FWC than fathers did (*b* = -0.221; *p* < 0.05), whereas there are no differences in WFC between mothers and fathers. This finding confirms Hypothesis 4, that fathers should experience higher levels of FWC than mothers do.

#### 4.2.3 Family Demands

To further disentangle whether there are differences among and between fathers and mothers in relation to childcare responsibilities, we looked at a categorical variable that combined the information on parents’ gender and childcare demands (supervision of preschool children and of schoolwork). As shown in Table 6, fathers with preschool children at home experience higher levels of FWC than did fathers without preschool children at home (*b* = 0.360; *p* < 0.05), but there is no such difference among mothers. Moreover, mothers show lower conflicts compared with fathers who had preschool children at home. Regarding schoolwork, mothers who engaged in supervision and those who did not show lower levels of FWC compared with fathers who engaged in this childcare activity. However, no intra-gender differences are detected, meaning that fathers’ and mothers’ FWC do not differ with respect to this family demand. Furthermore, we find no significant differences in WFC experiences in terms of varying childcare demands.

**TABLE 6:** Linear regressions on fathers’ and mothers’ FWC and WFC accounting for family demands (*N* = 227).

	Preschool children at home				Supervision schoolwork			
	FWC		WFC		FWC		WFC	
	Baseline	Interaction	Baseline	Interaction	Baseline	Interaction	Baseline	Interaction
Mothers	−0.283*		−0.173		−0.302*		−0.180	
	(0.126)		(0.167)		(0.135)		(0.166)	
Preschool children	0.316*		0.167					
	(0.135)		(0.135)					
Supervision schoolwork					0.094		0.096	
					(0.097)		(0.116)	
Gender and family demands								
(Ref. Fathers no preschool children)								
Fathers with preschool children		0.360*		0.237				
		(0.166)		(0.164)				
Mothers no preschool children		−0.241^a^		−0.107				
		(0.146)		(0.185)				
Mothers with preschool children		−0.016^a^		−0.085				
		(0.150)		(0.244)				
(Ref. Fathers no supervision schoolwork)								
Fathers with supervision schoolwork						0.198		0.180
						(0.136)		(0.146)
Mothers no supervision schoolwork						−0.147^a^		−0.055
						(0.149)		(0.176)
Mothers with supervision schoolwork						−0.248^a^		−0.115
						(0.163)		(0.204)
Constant	2.836*	2.783*	3.416*	3.323*	3.383**	3.334**	3.650**	3.610**
	(1.080)	(1.103)	(1.301)	(1.303)	(1.148)	(1.136)	(1.293)	(1.280)

Note: Controlled for age, partner, age of the youngest child, monthly gross income (log.), supervisory position, university degree, contracted working hours, experience in working from home; regressions on FWC during the pandemic control for pre-pandemic FWC, regressions on WFC during the pandemic control for pre-pandemic WFC; standard errors in parentheses; ^+^ *p* < 0.10, *p < 0.05, **p < 0.01, ***p < 0.001.

a Statistically different to fathers with childcare demands (preschool children at home or supervision schoolwork).

#### 4.2.4 Work Demands and Resources

In the next step, we analyze whether work demands and resources had different effects on the conflict experiences of parents and childless individuals (Hypothesis 5). The results shown in Table 7 indicate that working from home (*b* = -0.181; *p* ≤ 0.05), job autonomy (*b* = −0.034; *p* < 0.05), and family-supportiveness by co-workers (*b* = −0.259; *p* < 0.01) and supervisors (*b* = −0.139; *p* < 0.05) are all significantly related to lower WFC experiences during the COVID-19 pandemic, whereas increased overtime is related to higher WFC experiences (*b* = 0.368; *p* < 0.05). Yet none of the interactions between those job resources/demands and parenthood status are statistically significant, meaning that parents do not benefit more from resources and are no more impeded by demands located in the work sphere than did non-parents. Rather, such job resources are relevant for all employees in avoiding WFC to a similar degree and increased overtime is associated with more WFC for all employees as well.^9^ Consequently, Hypotheses 5 and 6a through 6c must be rejected for WFC.

**TABLE 7:** Linear regressions on employees’ FWC and WFC accounting for work resources and demands (*N* = 660).

	FWC					WFC				
	Increased overtime	Working from home	Job autonomy	Family-supportive-ness supervisor	Family-supportive-ness co-workers	Increased overtime	Working from home	Job autonomy	Family-supportive-ness supervisor	Family-supportive-ness co-workers
Women	−0.093	−0.096^+^	−0.095^+^	−0.116*	−0.099^+^	0.060	0.066	0.080	0.049	0.056
	(0.058)	(0.057)	(0.055)	(0.056)	(0.057)	(0.089)	(0.087)	(0.089)	(0.090)	(0.087)
Parents	0.182	0.044	0.103	0.226^+^	0.304**	0.226	0.139	0.094	0.336*	0.339*
	(0.113)	(0.145)	(0.221)	(0.117)	(0.115)	(0.137)	(0.186)	(0.356)	(0.148)	(0.149)
Work demands and resources										
Increased overtime	−0.036					0.368*				
	(0.107)					(0.150)				
Working from home		−0.056					−0.181*			
		(0.061)					(0.088)			
Job autonomy			−0.013					−0.034*		
			(0.013)					(0.016)		
Family-supportiveness supervisor				−0.049					−0.139^+^	
				(0.062)					(0.077)	
Family-supportiveness Co-workers					−0.142*					−0.259**
					(0.062)					(0.080)
Two-way interactions										
Increased overtime x Parents	0.142					0.028				
	(0.183)					(0.226)				
Working from home x Parents		0.221^+^					0.152			
		(0.123)					(0.168)			
Job autonomy x parents			0.012					0.020		
			(0.020)					(0.035)		
Family- supportiveness supervisor x parents				0.063					0.164	
				(0.101)					(0.135)	
Family- supportiveness Co-workers x parents					0.197^+^					0.185
					(0.114)					(0.138)
Constant	2.557***	2.608***	2.624***	2.897***	2.733***	1.438^+^	1.709*	1.891*	1.942*	1.880*
	(0.547)	(0.552)	(0.573)	(0.607)	(0.575)	(0.774)	(0.790)	(0.798)	(0.803)	(0.781)

Note: Controlled for age, partner, age of the youngest child, monthly gross income (log.), supervisory position, university degree, contracted working hours, experience in working from home; regressions on FWC during the pandemic control for pre-pandemic FWC, regressions on WFC during the pandemic control for pre-pandemic WFC; standard errors in parentheses; ^+^ *p* < 0.10, *p < 0.05, **p < 0.01, ***p < 0.001.

**FIGURE 3:**
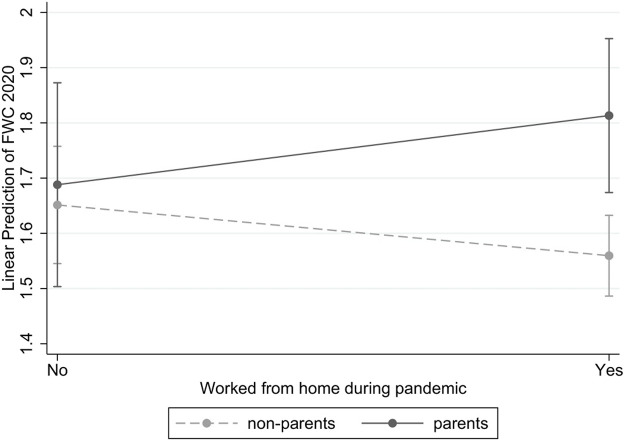
Relationship between Parenthood Status and Working from Home during the Pandemic on Parents and Non-Parents FWC. Note: Results based on Model M1, in Table 7. 90% confidence bands.

However, if we look at FWC, the results imply that not all employees can use working from home as a resource to achieve better integration of their family and work lives, as indicated by the significant interaction effect. Figure 3 displays the relationship between parenthood status and working from home during the COVID-19 pandemic on employees’ experience of FWC. The figure shows no significant differences in non-parents’ and parents’ FWC if they execute their work at the workplace. Yet parents who work from home experience significantly higher levels of FWC than their childless counterparts did (*b* = 0.221; *p* ≤ 0.10). Therefore, working from home seems to disadvantage parents and increase their strain. Nevertheless, we do not detect any differences among parents or non-parents who either did or did not work from home. Consequently, Hypothesis 6a is rejected, since parents do not benefit more than non-parents from this work resource. Furthermore, Hypothesis 6b is not supported by our results, as indicated by the non-significant interaction effect between job autonomy and parenthood status.

**FIGURE 4:**
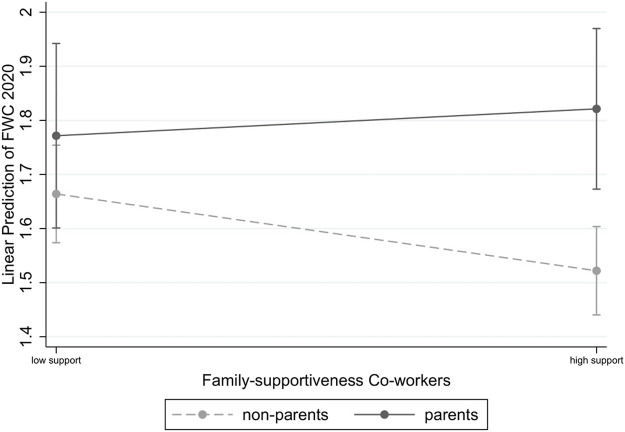
Relationship between Parenthood Status and Co-workers Family-supportiveness on Parents and Non-Parents FWC. Note: Results based on Model M4, in Table 7. 90% confidence bands.

Moreover, the estimates indicate that parents and non-parents are differently affected by the family-supportiveness of their co-workers with regard to their experienced FWC (*b* = 0.197; *p* < 0.10). As shown in Figure 4, parents and non-parents experience the same level of FWC if their co-workers are less family supportive. We detect significant differences in parents’ and non-parents’ FWC if their co-workers exhibit a high degree of family-supportiveness. Contrary to our assumption, however, parents have significantly more FWC experiences than did non-parents. Hence, Hypothesis 6c is likewise not supported by our results, since parents do not benefit to a greater extent than non-parents did from high degrees of family-support from co-workers.

## 5 Discussion

In addition to the obvious health threats posed by the COVID-19 pandemic, its manifold social consequences have drawn both scientific and societal interest. In our research, we specifically aimed to contribute to knowledge concerning gender and parenthood differences in work–life integration during the pandemic. To this end, we investigated both WFC and FWC and controlled for the situation before the pandemic. Only then the results we find can be meaningfully interpreted as induced by the COVID-19 pandemic. Moreover, we tested competing expectations about the direction in which the pre-pandemic situation might influence conflict experiences during the pandemic.

Our first major finding was that work–family conflicts did indeed increase during the COVID-19 pandemic, but that this applied only to FWC. Second, our findings support the idea that over and above being a control variable, previous conflict experiences are substantively important for experiences of WFC and FWC during the pandemic. For WFC, we found support for the accentuation hypothesis, suggesting that higher levels of conflicts will be accentuated during the stressful situation of the pandemic. This finding supports assumptions on cumulative disadvantages (Crystal and Shea, 1990; O’Rand, 1996)—that is, previously disadvantaged individuals (in terms of a higher WFC) are at higher risk for disadvantages with regard to WFC during the COVID-19 pandemic. As an alternative theoretical perspective we asserted that exposure to limited adverse experiences may foster subsequent resilience toward future adversity, since exposed individuals would acquire coping strategies and learn to handle those situations more effectively (Seery et al., 2010). However, those learning processes might not be successful if the experienced strain is either too low or too high, suggesting a U-shaped relationship between previous adversity experiences and current experiences (Seery, 2011; Seery et al., 2013). Indeed, we found such a non-linear U-shaped relationship for FWC, although it was in the opposite direction. It was only if the levels of FWC before the pandemic exceeded a certain threshold that individuals were able to learn from previous conflict experiences and experienced lower levels of conflicts during the COVID-19 pandemic.

Third, looking at gender and parenthood revealed that parents experienced more FWC than did non-parents, and that among parents, fathers in particular experienced significantly intensified conflicts in the family-to-work direction. For WFC, we did not find significant differences in conflicts at all when we controlled for pre-pandemic conflict levels. These results partly support previous results for Germany (Buschmeyer et al., 2021), although only for FWC, but they contradict studies elsewhere that even found decreases in WFC during the COVID-19 pandemic (Schieman et al., 2021). However, our results add to those of previous studies by showing that there were no overall gender differences in WFC and FWC experiences during the pandemic, but that among parents, gender did play a role. Fathers, in particular, struggled with the challenge of balancing family and work responsibilities during the pandemic, especially when they needed to compensate for the lack of childcare by caring for their preschoolers or schoolchildren at home. Here, working from home played an important role and becomes relevant for explaining FWC during the pandemic. This finding contributes to previous research concerning the antecedents of different directions of conflicts, which showed that, similar to most job demands and resources, working from home was mostly relevant for WFC perceptions before the pandemic (Allen et al., 2013). During the pandemic, working from home likewise predicted FWC, which seems reasonable since boundaries had become much more blurred.

Our study also contributes to the vivid debate on working from home. The results emphasize that working from home is more conflictual for parents than for childless individuals, since parents experienced even higher levels of FWC when working from home during the COVID-19 pandemic. According to the existing literature, working from home is ambivalent in its effect on reconciling work and family responsibilities, results being mixed with regard to whether it increases or decreases stress and effective reconciliation (Allen et al., 2015). Under the lockdown conditions of the pandemic, the pendulum seems to swing to the negative side, obviously mostly as a result of the extraordinary, very demanding childcare situation that is also related to more FWC. However, as specific as the circumstances are, these results should not be too easily generalized when it comes to the potential effects of working from home under largely different conditions. Still, our findings provide some hints about the conditions under which working at home will not be an advantage. Especially when having young children, parents still need external childcare to successfully integrate their work and family life responsibilities even if they can work from home.

Our results regarding family-supportiveness by co-workers were quite counterintuitive. Non-parents perceived lower levels of FWC when their co-workers were highly supportive, whereas parents experienced even higher levels of FWC when they received high levels of support. A speculative explanation of these findings could be that if parents work in a team that is characterized by mutual understanding and a supportive atmosphere, they may feel obligated to return some of the support that they receive from others. However, not being able to return such support because of overwhelming work and family demands might ultimately result in more conflict experiences.

Increased levels of FWC during the COVID-19 pandemic does not affect parents only. We found that childless individuals also experienced an increase in FWC, especially men who were childless. Therefore, not only parents were disadvantaged in their work–life integration during the pandemic, but individuals without children also struggled with this stressful situation. A possible explanation might be that resources for compensating work and family strain were limited owing to contact restrictions and fewer opportunities for leisure activities. For instance, social support by friends or by co-workers is known to be an important resource for mitigating both WFC and FWC experiences (Carlson and Perrewé, 1999; Reimann et al., 2019). Social interactions, however, are negatively affected by the containment measures of social distancing. This may particularly apply to childless individuals who have fewer possibilities for social interactions within their own households. Moreover, our results indicate that although it is essential to consider the heterogeneity of the workforce, some key conditions benefit all employees when it comes to better integrating work and family demands. Being able to work from home during the pandemic, having greater job autonomy, and working in a family-supportive work environment all predicted lower WFC for all employees. This again underscores that it is not only parents who face difficulties in combining work and family demands; non-parents likewise face reconciliation conflicts where, for instance, family-supportive work environments reduce experiences of WFC. Thus, if employers aim to promote better work–family integration, strengthening these working conditions might be a starting point.

The results of this study have some limitations. First, we conducted cross-sectional analyses using the COVID-19 follow-up survey of the LEEP-B3 data. Though we accounted for WFC and FWC prior to the COVID-19 pandemic by using information from previous waves of the panel data, assumptions with regard to the causal directions of the relationships should be made with caution. Second, the results of our analyses refer to a sample of workplaces with more than 500 employees. These employees are on average better protected than those in smaller workplaces, as reflected in the very low representation of employees with reduced working hours during the pandemic. Our sample might also be biased because of possible selectivity when recruiting participants for the follow-up survey. For instance, there may have been selectivity due to work–family conflicts themselves, since individuals with extremely high levels of conflicts might not have participated in the survey at all because of their high stress levels. However, this seems to be a minor issue, since the selectivity analyses we carried out did show that parents were not less likely to participate even though they had to manage higher demands. Third, our study lacks additional information about the private life situations of parents (e.g., regarding the exact childcare arrangements within partnerships during the pandemic and regarding other possibilities for childcare support), but also of childless individuals, especially regarding their leisure activities and social relationships other than partnerships. Especially the lack of information on additional childcare possibilities during the pandemic is an important drawback of our research. However, as external childcare was restricted due to the closing of childcare facilities and informal childcare by family or friends outside the direct household context was also very limited to the rigorous contact restrictions, it seems most likely that additional childcare opportunities were only minorly important during the COVID-19 situation (Collins et al., 2020; Kohlrausch and Zucco, 2020; Möhring et al., 2020; Müller et al., 2020; Zoch et al., 2021b). Since the results clearly indicate that FWC has gained importance during the COVID-19 pandemic, future research should focus more on changes in the family and private life sphere to further explain differences in FWC experiences.

Despite these limitations, we provide important insights into the overall picture of how men and women and mothers and fathers have been affected by the extra strains imposed by the pandemic, over and above the narrow work–family conflict perspective. As mentioned above, there is little doubt that it was mainly women and particularly mothers rather than fathers who had to shoulder more of the added care demands. Still, fathers also took over some part of these additional burdens. However, from the work–family conflict perspective, fathers experienced higher levels of FWC than mothers did. Apparently, there is no simple equation that more strains cause more conflict. More research is needed to solve this puzzle. Did fathers experience comparably more FWC because they received less understanding from their employers, supervisors, or co-workers, or were they trapped because they had fewer experiences in simultaneously handling family and work demands, or did other within-household bargaining processes determine fathers’ higher levels of FWC? We cannot fully answer this question, but our results point more toward processes at the household level, since we controlled for a wide range of workplace conditions in our analyses.

## Data Availability

The data analyzed in this study is subject to the following licenses/restrictions: The datasets analyzed during the current study are not publicly available due to data restrictions by the Federal Institute for Employment Research (Institut für Arbeitsmarkt und Berufsforschung (IAB). Data are only available on request for analyses to be conducted locally at Bielefeld University in cooperation with project members. Requests to access these datasets should be directed to martin.diewald@uni-bielefeld.de.

## Footnotes

1 Work–family conflicts are also referred to as work–life conflicts in the literature. For consistency we use the term work–family conflicts but explicitly emphasize that conflicts can be important experiences for all individuals, not just for parents or couples. When using this term, we also refer to the overall concept of work–family conflicts, including the directions of family-to-work and work-to-family conflicts.

2 Employees earning more than 450€ per month.

3 Of all employees in Germany, 19.1% work in these establishments, which make up 0.2% of all German workplaces (Frodermann et al., 2018).

4 The former question represents the strain-based dimension of FWC and the latter question the time-based dimension (e.g., Carlson et al., 2000). However, since we were interested in overall family–work conflicts during the pandemic, these two items were summed and divided by 2; the same holds for overall WFC.

5 Selectivity analyses reveal that men, higher educated employees, older employees, non-supervisors, and workers in the credit and insurance sectors were more likely to have participated in the COVID-19 follow-up survey (see Supplementary Appendix Table SA1). Nevertheless, effects were relatively low except for the highest educated employees, who were 12.5% more likely to have participated than the least educated ones.

6 “Less supportive” was defined as all answers lower than 4 (“Applies completely”).

7 Childless employees likewise stated that they faced these family demands. This was the case because employees with older children were defined as childless. Hence, it could be that some parents were grandparents and took care of their grandchildren during the pandemic.

8 Not accounting for this nested data structure in which employees work in the same establishment would lead to too low standard errors and thus falsely significant results (Hox, 2010). Hence, applying robust standard errors produces more conservative estimates.

9 Models estimating the direct effects of resources and demands on FWC and WFC during the pandemic (without the group interactions) underline this finding (see Supplemantary Appendix Table SA2).
