# Supplementary material for: COVID-19 and Work–Family Conflicts in Germany: Risks and Chances Across Gender and Parenthood
Source: Front Sociol. 2022 Jan 5;6:780740. doi: 10.3389/fsoc.2021.780740 (PMC8767121; doi:10.3389/fsoc.2021.780740)
Supplement: Supplementary file 1 [file Table1.docx]

**Supplementary Appendix**

**Table SA1.** Logistic Regressions on the Probability to have Participated in the LEEP-B3 Follow-Up Survey (Average Marginal Effects)

|  | **Selectivity Analysis**  (*N***=**5,150) |
| --- | --- |
| Women | -0.028^*^ |
|  | (0.011) |
| Parents | -0.011 |
|  | (0.010) |
| Age of the Youngest Child | -0.001 |
|  | (0.007) |
| Age in Years | 0.003^*^ |
|  | (0.011) |
| Tenure in Years | 0.000 |
|  | (0.000) |
| Experience in Years | 0.000 |
|  | (0.000) |
| Education (*Ref. Low Educational Qualifications*) |  |
| Intermediate | 0.057^***^ |
|  | (0.012) |
| High | 0.125^***^ |
|  | (0061) |
| Monthly Gross Income | 0.000^***^ |
|  | (0.000) |
| Industry Sector (*Ref. Manufacturing*) |  |
| Retail, Hospitality, and Transportation | -0.015 |
|  | (0.020) |
| Financial and Insurance Services | 0.027^*^ |
|  | (0.012) |
| Education, Health, and Public Services | -0.010 |
|  | (0.135) |
| Constant | -0.001^***^ |
|  | (0.301) |
| Note: Standard errors in parentheses: ^+^ *p* < 0.10 ^*^ *p* < 0.05, ^**^ *p* < 0.01, ^***^ *p* < 0.001 | |

**Table SA2**. Linear Regressions on Pre-COVID-19 Work-Life Conflicts and Work-Life Conflicts during the Pandemic Accounting for Non-Linearity

(*N*=660)

|  | FWC  (M1) | WFC  (M2) |
| --- | --- | --- |
|  |  |  |
| ***Gender & Parenthood*** *(Ref. Fathers*) |  |  |
| Childless Men | -0.343^**^ | -0.251^+^ |
|  | (0.118) | (0.128) |
| Childless Women | -0.409^**^ | -0.201 |
|  | (0.125) | (0.144) |
| Mothers | -0.199^*^ | -0.009 |
|  | (0.098) | (0.156) |
| FWC 2018/19 | 0.941^***^ |  |
|  | (0.149) |  |
| FWC 2018/19² | -0.132^***^ |  |
|  | (0.036) |  |
| WFC 2018/19 |  | 0.600^***^ |
|  |  | (0.177) |
| WFC 2018/19² |  | -0.013 |
|  |  | (0.031) |
| Constant | 2.636^***^ | 1.989^*^ |
|  | (0.595) | (0.858) |
| Note: Controlled for age, partner, age of the youngest child, monthly gross income (log.), supervisory position, university degree, contracted working hours, experience in working from home.  Standard errors in parentheses; ^+^ *p* < 0.10, ^*^ *p* < 0.05, ^**^ *p* < 0.01, ^***^ *p* < 0.001 | | |
